# Supplementary material for: From sequence to enzyme mechanism using multi-label machine learning
Source: BMC Bioinformatics. 2014 May 19;15:150. doi: 10.1186/1471-2105-15-150 (PMC4229970; doi:10.1186/1471-2105-15-150)
Supplement: Additional file 2 — Java code of ml2db. Additional file ml2db_code.tar.gz contains the Java source code to run the multi-label machine learning experiments and save the results to database. The code’s Javadoc is included. [file 1471-2105-15-150-S2.zip › additional file 2/ml2db/utils/doc/uk/ac/ed/inf/utils/database/class-use/DbManager.html]

Uses of Class uk.ac.ed.inf.utils.database.DbManager


---


|  |  |  |
| --- | --- | --- |
| **Overview** \| **Package** \| **Class** \| **Use** \| **Tree** \| **Deprecated** \| **Index** \| **Help** | |  |
| PREV   NEXT | **FRAMES**    **NO FRAMES**     **All Classes** |  |


---


## **Uses of Class uk.ac.ed.inf.utils.database.DbManager**

| Packages that use DbManager | |
| --- | --- |
| **test.database** |  |
| **uk.ac.ed.inf.utils.collectionutils.setutils** |  |
| **uk.ac.ed.inf.utils.database** |  |
| **uk.ac.ed.inf.utils.setutils** |  |

Uses of DbManager in test.database

| Methods in test.database that return DbManager | |
| --- | --- |
| `static DbManager` | `DbManagerTest.getDataDbManager()` |
| `static DbManager` | `DbManagerTest.getDbManager()` |

Uses of DbManager in uk.ac.ed.inf.utils.collectionutils.setutils

Constructors in uk.ac.ed.inf.utils.collectionutils.setutils with parameters of type DbManager

`SupersetsManager(java.lang.String sqlQuery,
DbManager dbManager,
java.lang.String separator)`

Uses of DbManager in uk.ac.ed.inf.utils.database

| Methods in uk.ac.ed.inf.utils.database that return DbManager | |
| --- | --- |
| `DbManager` | `Table.getDbManager()` |
| `DbManager` | `DbManaged.getDbManager()` |

| Methods in uk.ac.ed.inf.utils.database with parameters of type DbManager | |
| --- | --- |
| `TableManager` | `Table.getTableManager(DbManager manager)` |

Constructors in uk.ac.ed.inf.utils.database with parameters of type DbManager

`DbCreator(DbManager dbManager)`

`DbManaged(DbManager dbManager)`

`DbManager(DbManager dbManager)`
  
          Constructor 2: creates the database manager from another one

`DbReader(DbManager dbManager)`

`DbWriter(DbManager dbManager)`

`Table(java.lang.String tableName,
java.util.Vector<TableColumn> columns,
DbManager dbManager)`

`TableManaged(DbManager dbManager,
TableManager tableManager)`

`TableManager(DbManager dbManager,
java.lang.String tableName)`
  
          Constructor to create a generic manager to read/write to a table.

`TableManager(DbManager dbManager,
Table table)`
  
          Constructor to create a manager to create, read and write a table.

Uses of DbManager in uk.ac.ed.inf.utils.setutils

Constructors in uk.ac.ed.inf.utils.setutils with parameters of type DbManager

`SupersetsManager(java.lang.String sqlQuery,
DbManager dbManager,
java.lang.String separator)`

---


|  |  |  |
| --- | --- | --- |
| **Overview** \| **Package** \| **Class** \| **Use** \| **Tree** \| **Deprecated** \| **Index** \| **Help** | |  |
| PREV   NEXT | **FRAMES**    **NO FRAMES**     **All Classes** |  |


---
